# Supplementary material for: Theta oscillations show impaired interference detection in older adults during selective memory retrieval
Source: Sci Rep. 2019 Jul 10;9:9977. doi: 10.1038/s41598-019-46214-8 (PMC6620337; doi:10.1038/s41598-019-46214-8)
Supplement: Supplementary file 1 — Supplementary Material [file 41598_2019_46214_MOESM1_ESM.pdf]

Theta oscillations show impaired interference detection in older adults during selective memory retrieval

Catarina S. Ferreira[1, 2*], Maria J. Maraver[1, 3], Simon Hanslmayr[2] and Teresa Bajo[1]

[1]Research Center for Mind, Brain and Behaviour, University of Granada, Spain

[2]School of Psychology, University of Birmingham, United Kingdom

[3]Cognitive Psychology Unit & Leiden Institute for Brain and Cognition, Leiden University, The Netherlands

*Correspondence concerning this article should be addressed to Catarina S. Ferreira, School of Psychology, University of Birmingham, Edgbaston, Birmingham, B15 2TT (United Kingdom). E-mail: a.c.sanchesferreira@bham.ac.uk

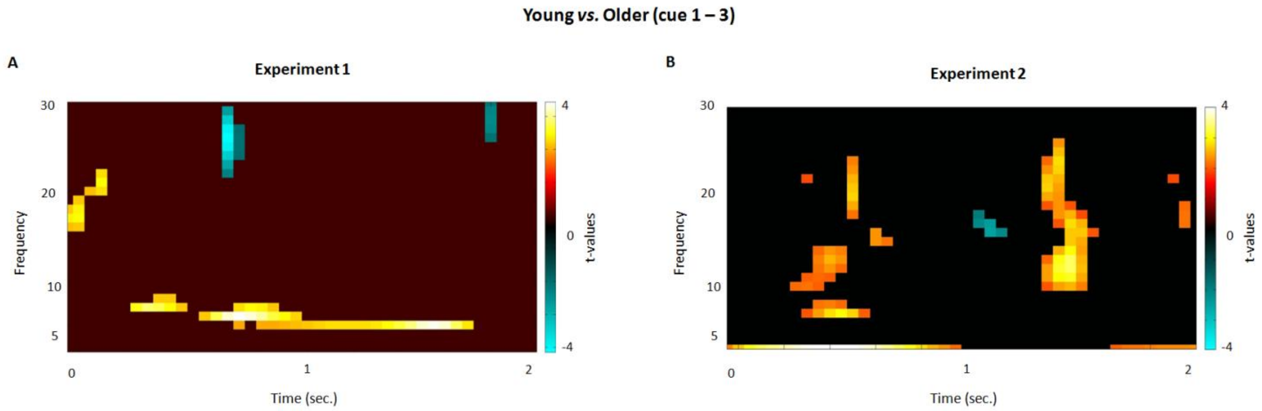

Supplementary Figure 1: Interaction analysis: time-frequency plots of the differences between younger (cue1–cue3) and older adults (cue1 – cue3), over all electrodes and for a broader time (0 to 2 sec.) and frequency (0 to 30 Hz) ranges. A) depicts the time-frequency plot obtained from the data of Experiment 1 and B) from Experiment 2.

Supplementary Table 1: Average number of valid trials after pre-processing (Mean(SD)) for each retrieval cue, age group and experiment.

	Experiment 1		Experiment 2	
	Young	Old	Young	Old
Cue 1	21.54 (1.72)	20.00 (2.62)	22.21 (1.72)	22.48 (1.38)
Cue 2	22.04 (2.01)	20.40 (2.09)	22.29 (1.49)	23.17 (0.83)
Cue 3	22.29 (1.37)	20.40 (2.82)	22.38 (1.31)	23.04 (1.40)
